# Supplementary material for: Are carbon emissions trading and green financial instruments synergistic? -Comprehensive quantitative research based on content analysis
Source: PLoS One. 2024 Mar 7;19(3):e0298601. doi: 10.1371/journal.pone.0298601 (PMC10919601; doi:10.1371/journal.pone.0298601)
Supplement: S1 Data — (ZIP) [file pone.0298601.s001.zip › Data/地级市Z_F工作报告.zip!/┴╔─■/╙¬┐┌╩╨/└┤╫╘╣1⁄2╓┌║┼í╛┬φ┐╦╩2╛▌═°í┐_2006.docx]

各位代表：

　　我代表市人民政府向大会报告工作，请予审议，并请政协委员和列席会议的同志提出意见。

　　一、２００５年工作的回顾

　　２００５年，在市委的正确领导下，我们以邓小平理论和“三个代表”重要思想为指导，以加速老工业基地振兴、构建和谐营口为主题，用科学发展观统领经济社会发展全局，国民经济和社会事业实现了快速、健康发展。全市完成地区生产总值３８０．９亿元，比上年增长２０．６％；全社会固定资产投资２２１．２亿元，增长４０％；社会消费品零售总额１０７亿元，增长１４．６％；城市人均可支配收入９００９元，增长１０．８％；农民人均纯收入４５０３元，增长１６．４％。

　　切实加强“三农”工作，农业和农村经济稳步发展。全年完成第一产业增加值４１亿元，增长９．５％。粮食总产量６７．５万吨，增长１１．６％，水果、水产品、蔬菜、畜产品产量稳步提高。新建、扩建投资５００万元以上农业产业化项目２０个，总投资达５．５亿元。完成农用地环评２２５万亩，推进了农产品无公害基地建设和市场准入制度试点工作。新建灌溉工程２６２处，治理水土流失面积８．７万亩。完成人工造林６．４万亩，植树１６０５万株。加强无规定动物疫病区建设，有效防止了高致病性禽流感疫情的发生。全年免征农业税２８７７万元，取消不合理涉农收费２０５万元。

　　大力推进体制创新和结构调整，工业经济实现速度和效益同步增长。全市第二产业增加值达到１９５．４亿元，增长２９．１％；规模以上工业总产值５４０．９亿元，增长４０．４％；利税总额３８．１亿元，增长２５％。实施国有企业改制５４户，全面完成省下达的改革任务。民营经济在改革调整中快速发展，实现企业增加值２４９．６亿元，增长２４．１％，占地区生产总值比重达到６５．５％。实施５００万元以上技改项目１５４项，开发新产品７５５种，完成工业固定资产投资１４３．７亿元，增长７１．３％。全市省以上名牌产品达到３２种，盼盼集团、三征公司分别荣获中国名牌产品和中国驰名商标，名牌牵动作用显著增强。

　　积极开展招商引资活动，对外开放取得丰硕成果。全年新批外商投资企业１５８家，实际利用外资２．４亿美元，增长４１％；引进内资８５．２亿元，增长１９．６％。开工建设投资５００万元以上项目５４８项，其中５０００万元以上１０９项。完成进出口总额１５．１亿美元，增长２７．９％，其中出口１０．２亿美元，增长３０．３％。先导区作用不断增强，开发区实际利用外资９３００万美元，增长２８％；出口创汇４．６亿美元，增长３０％。高新区新开工５００万元以上项目３３个，实现技工贸总收入２２．４亿元，增长１倍。港口建设取得突破性进展，一港池油品及液体化工码头等工程顺利投产，全年完成吞吐量７５３７万吨，增长２６．１％；集装箱运量７８．７万标箱，增长３５％。辽宁（营口）沿海产业基地列入省政府“十一五”规划，一期４０平方公里土地整理工程已全面展开。

　　加强城市建设和管理，城市综合功能明显提升。棚户区改造取得历史性突破，全年实施棚户区改造项目１７个，拆迁占地面积１３３．８万平方米，回迁楼开工面积达５２．３万平方米。重点工程得到全面实施，滨海热电厂、西部污水处理一期、市区供水管网改造一期、中水回用土建工程等项目如期完成；取缔污染严重的小锅炉４２座，新增集中供热面积１８４万平方米。年初确定为市民办的１５件实事已完成１０件，其余５件跨年度工程正按计划实施。全年出让土地使用权２６宗，成交金额９．５亿元。城市综合治理效果明显，亮化、净化、绿化和美化水平得到提高，大气质量保持在国家二级水平。

　　切实做好财税金融工作，宏观调控能力逐步增强。继续深化部门预算、集中支付等项改革，加强税收征管，努力打造“阳光财政”。全市财政一般预算收入完成１６．２亿元，增长２５．９％。金融机构各项存款余额４０２亿元，增长１８．３％；各项贷款余额２６２亿元，增长１３．５％。商业性保费收入９．２亿元，增长２３．５％。不良贷款比率比年初下降１８．６个百分点，金融安全区建设目标基本实现。政府出资引导组建了中小企业信用担保公司，支持城市商业银行完成了不良贷款置换，农村信用社在全省率先完成增资扩股任务。

　　强化就业和扶贫帮困工作，社会保障体系不断完善。全年新增实名制就业８．８２万人，登记失业率控制在５．２％，在全省提前半年率先解决了“零就业家庭”的就业问题。培训下岗失业人员４．１万人，培训后就业率达６１．９％。新建农村剩余劳动力培训基地１２个，转移农村剩余劳动力３．８万人。企业养老保险新增扩面５．１９万人，医疗保险新增参保人员５万人。对６．８万名城市困难居民实行最低生活保障，并对重点低保对象实施了分类救助。全年共接收社会捐赠款物１１１７万元，救助城乡贫困群众３．０５万户。

　　协调发展社会各项事业，精神文明建设得到加强。积极推进科技进步与创新，申报省级以上科技项目８２项，引进和推广科技新品种４２个、新技术１３项，高新技术产业增加值增长３３％。教育事业发展较快，高考、中考工作取得新成绩，“双高普九”工作扎实推进，职业技术教育招生和就业工作取得新成效。教育基础设施建设步伐加快，农村中小学危房改造进展顺利，消灭了现存Ｄ级危房。城乡文化生活进一步活跃，成功举办了母亲节等重大节庆文艺演出。文化基础设施不断完善，文物保护工作受到重视，文化市场秩序明显好转。广电事业取得新发展，开通了数字电视业务，实现了国家规定的广播电视节目全覆盖。城乡公共卫生体系建设步伐加快，新型农村合作医疗试点工作有序进行，医疗卫生资源整合与基础设施建设实现历史性突破。计划生育取得新成绩，人口自然增长率控制在１．１９‰。全民体育活动广泛开展，竞技体育水平有所提高。精神文明建设不断加强，公民精神面貌发生了新的变化。

　　加强民主和法制建设，政府依法行政水平进一步提高。自觉接受人大依法监督和政协民主监督，全年办理人大代表建议２０９件，政协提案２３４件，办复率１００％，满意率９９．６％。加强同民主党派、工商联、人民团体及社会各界人士的联系，全面落实党的民族、宗教和侨务政策。深入开展“四五”普法、“三五”依法治市工作，“法治营口”建设取得明显成效。全面推进警务改革，强力开展破案攻坚和治安综合治理，全市刑事案件发案率明显下降，被评为全省综合治理先进市。严格执行《信访条例》，实行重点案件领导包案制度，信访案件同比下降３０％。积极实施食品药品放心工程，强化安全生产责任制，避免了重特大事故的发生。认真贯彻《行政许可法》，切实加强了政风、行风建设，政务环境得到改善。国防教育和民兵预备役工作扎实推进，双拥成果得到巩固。

　　各位代表，２００５年是实施“十五”计划的最后一年。过去的五年，我们始终坚持以发展为第一要务，积极弘扬昂扬向上的发展气势和敢为人先的创新精神，经受了经济体制转轨的重大考验，战胜了各种自然灾害、非典和禽流感疫情的严峻挑战，有效抑制了经济运行中的各种不利因素，超额完成了“十五”计划任务。五年累计完成固定资产投资比“九五”期间增长２．４５倍。２００５年全市地区生产总值比２０００年增长１．０３倍，经济总量由全省第９位上升至第７位；财政一般预算收入增长２．７倍；港口吞吐量增长２．３２倍。过去的五年，我市综合竞争力显著增强，城乡人民生活水平得到改善，物质文明、政治文明和精神文明实现了全面发展。

　　这些成绩的取得，是市委正确领导的结果，是市人大、政协及社会各界、驻营各单位对政府工作监督、帮助、支持的结果，是全市人民团结奋斗、共同努力的结果。在此，我代表市人民政府，向全市广大干部、群众，向各位人民代表、政协委员，向驻营部队和中省直单位，向所有关心、支持营口建设的同志们、朋友们，表示崇高的敬意和衷心的感谢！

　　在肯定成绩的同时，我们也清醒地看到，全市经济和社会发展中仍然存在一定的困难和问题：一是农村经济市场化程度低，社会化服务相对滞后，生态建设和环境保护任务很重；二是工业经济结构性矛盾较为突出，高新技术产业发展缓慢，企业自主创新能力不强；三是利用外资的规模不大，高技术、高附加值的项目较少；四是地方财政供给不足，债务负担沉重，收支矛盾突出；五是社会保障和再就业压力增大，劳动就业结构性矛盾突出，部分群众生产生活仍很困难；六是政府职能和工作作风转变不够，少数干部缺乏大局意识和服务意识，为政不勤、为政不实、为政不廉的现象依然存在。对此，我们必须引起高度重视，在今后的工作中认真加以解决。

　　二、２００６年工作的主要任务

　　各位代表，２００６年是实施“十一五”规划的开局之年，做好今年的政府工作，意义十分重大。我们要按照省委和市委九届十一次全会的要求，进一步解放思想，抢抓机遇，加速推进营口经济的全面振兴。

　　２００６年市政府工作总的指导思想是：以邓小平理论和“三个代表”重要思想为指导，认真贯彻党的十六届三、四、五中全会和中央经济工作会议精神，坚持以科学发展观统揽全局，坚定不移地实施“工业强市、以港兴市”发展战略，以深化改革、扩大开放和科技进步为动力，以调整和优化结构为主线，以实现“沿海率先崛起”和城乡区域经济协调发展为目标，大力推进项目建设，着力改善发展环境，努力保持经济和社会持续、快速、健康发展，全面提高人民生活水平，积极构建和谐营口，为“十一五”规划的实施开好局、起好步。

　　２００６年经济社会发展的主要目标是：地区生产总值４５０亿元，比上年增长１８％；全社会固定资产投资２７６亿元，增长２５％；实际利用外资３．１２亿美元（按可比口径），增长３０％；出口创汇１２．７５亿美元，增长２５％；社会消费品零售总额１２１亿元，增长１３％；地方财政一般预算收入１９．５亿元（按可比口径），增长２０％；城市人均可支配收入１００９０元，增长１２％；农民人均纯收入４９５１元，增长１０％；人口出生率控制在６．１９‰；城镇登记失业率控制在５％。

　　为实现上述目标，要重点抓好以下十二个方面工作：

　　（一）认真贯彻党的十六届五中全会精神，确保“十一五”经济社会发展取得良好开局

　　全面落实科学发展观，努力实现可持续发展。按照“立足科学发展，着力自主创新，完善体制机制，促进社会和谐”的基本要求，坚持率先发展、科学发展、和谐发展，实行富民优先、科教优先、环保优先、节约优先的方针，在更高起点上推动经济社会平稳快速增长。到２０１０年，地区生产总值达到８８０亿元，年均增长１８％；全社会固定资产投资累计完成２１５０亿元，年均增长２５％；实际利用外资累计完成２８亿美元（按可比口径），年均增长３０％；地方一般预算收入达到４８亿元（按可比口径），年均增长２５％；城市居民人均可支配收入１５８７０元，年均增长１２％；农民人均纯收入７２５０元，年均增长１０％。

　　突出战略重点，把握发展大局。坚持总量扩张与转变增长方式相结合的原则，在做大经济总量的同时，进一步提高经济增长的质量和效益；坚持城乡协调发展的原则，充分发挥城市的带动作用，加快社会主义新农村建设；坚持人与自然和谐发展的原则，合理利用资源，加强环境保护，提升城乡环境质量；坚持深化改革与扩大开放相互促进的原则，以改革促开放、促调整、促发展；坚持着眼长远与立足当前相结合的原则，既要着力解决当前面临的突出矛盾和问题，又要统筹谋划好未来五年的经济社会发展，为营口的全面振兴奠定基础。

　　（二）深入开展“工业发展年”活动，做大做强工业经济

　　积极营造氛围，全力推进工业化进程。按照振兴营口老工业基地的总体要求，把“工业化”作为实现经济总量扩张、提高经济增长质量和推进小康社会建设的重要途径，以发展大工业为目标，通过构建中小企业服务体系、鼓励高技能劳动力输入、增加财政科技投入等措施，创造良好的政策环境，促进产业结构优化升级，提高工业经济运行质量和效益。

　　坚持以项目为纲，强化项目的支撑作用。进一步增强项目意识，建立完善项目推进机制和领导责任制，把全社会的各种智慧和力量凝聚到项目开发建设上。坚持大小项目并举的方针，加快鞍钢营口新区、五矿中板、重交沥青、钛白粉等重大项目推进，千方百计引进一批“专精特新”的中小项目，全年新开工建设投资１０００万元以上项目３００个，其中亿元以上８０个。

　　积极培育产业集群，调整优化工业结构。紧紧围绕“抓产品、上项目、壮企业、兴产业”的要求，制定和完善各类开发区和工业园区规划，加强基础设施建设，提高园区承载能力。精心打造冶金、石化、装备制造、镁质材料、纺织服装、新型建材六大产业集群，积极引进和培育关联性大、带动性强的大企业、大集团、大项目，促进中小企业群体的快速发展，形成骨干带动、产业密集、链条完善的产业体系。

　　进一步深化企业改革，提高企业整体素质。按照《营口市深化企业改革实施方案》，打好企业改革攻坚战，全年完成列入省考核范围的国有企业改制１０户，列入市考核范围的国有及集体企业改制８０户。要进一步鼓励支持民营企业发展，引导其参与国有和集体企业的改制和重组，促进民营企业的机制创新。

　　大力推进企业技术进步，增强企业自主创新能力。加大企业技术改造力度，积极引进和消化吸收国内外先进技术，全年实施重点技术改造项目１００项。加速培育科技先导型企业，新建企业技术中心１０个，提高企业研发能力。开发重点新产品２００种，实现新产品产值６０亿元。继续实施名牌战略，制定并实施奖励政策，加强对重点产品和骨干企业的扶持，积极培育和壮大品牌优势。

　　积极发展循环经济，建设节约型社会。加强矿产资源的保护、开发和有效利用，提高制成品和高附加值产品的比重。高度重视水资源的有效利用，加强水源地的环境保护。在冶金、石化、建材等重点行业推行资源循环利用发展模式，抓好６户重点污染企业清洁生产审核和２０个重点节能项目实施，实现工业经济的可持续发展。全年万元地区生产总值综合能耗下降４％，以提高环境承载力为基础，大力开展水、土地、能源等的综合利用，积极倡导建设环境友好企业和争做环境友好公民。

　　（三）大力发展现代农业，加快社会主义新农村建设

　　不断优化农业结构，促进优势产业发展。以“三水一牧一菜”为重点，推动农业向高产、优质、高效、生态、安全方向发展。严格落实耕地保护政策，粮食作物生产面积稳定在１４０万亩。提高水产业精养水平，全年工厂化养殖面积达到２３万平方米。正确处理林畜矛盾，加快畜牧饲养小区、饲养场建设，畜牧业产值增长１０％以上。推进盖州国家无公害果品示范基地建设，努力提高果品质量。积极推广农业标准化，全年新认定无公害生产基地１０个、无公害生产品种２０个。建立和完善柞蚕、水果等１０个农产品出口生产基地，农产品出口创汇达到９０００万美元，增长１２．５％。

　　加大财政对农业的投入力度，促进农村经济社会协调发展。高度重视“三农”问题，认真落实各项惠农政策，确保各级财政对农业的投入增长１８％以上。全年新建续建农业产业化龙头项目２０个，兴建大型综合市场６个。搞好以库塘井窖为重点的农田基本建设，积极推广农业机械化，增强农业综合实力。大力发展生态经济林，全年完成造林４万亩，植树８００万株。强化小城镇和村屯的规划、管理，完善交通、供水、供电等基础设施，加快发展教育、文化、卫生等公共事业，提高农村城镇化水平。

　　继续深化农村改革，完善农村社会化服务体系。认真贯彻《农村土地承包法》，促进土地向规模经营方向发展。推进集体林产权制度改革，促进森林资源的培育、保护和生态建设。深化农村金融体制改革，构建农村信用担保体系。加快建设农业信息服务网络，建立健全各类专业合作经济组织和经纪人队伍，提高农民进入市场的组织化程度。坚决制止不合理收费，切实减轻农民负担。

　　（四）积极扩大外引内联，努力提高对外开放水平

　　全力拓展招商领域和渠道，进一步加大招商引资力度。围绕加快沿海产业基地建设、发展区域特色经济和满足境内外投资者的需求，策划包装１００个市场竞争力强的大项目，强化对重点国家、地区及重点企业外资项目的服务和推进。不断创新招商方式，完善招商政策，建立完善以企业为主体、政府为指导、专业队伍为骨干、中介组织为辅助的招商机制，提高招商引资的成功率。加强与辽宁中部城市群和环渤海地区合作，主动承接国内发达地区产业梯次转移。全年组织内联招商团组１００个，落实投资５００万元以上内联项目２００项，力争引进内资１００亿元。

　　转变对外贸易增长方式，努力实现出口新增长。鼓励企业调整产品结构，扩大拥有自主知识产权和自主品牌的产品出口。充分利用中小企业国际市场开拓资金和振兴东北老工业基地外贸资金，确保各项鼓励出口政策落实到位。组织企业参加各类商品交易会和博览会，寻求出口商机。积极参与商品配额招投标工作，扩大纺织品和镁制品的出口份额。发挥行业协会、商会的自律和协调作用，规范企业行为，有效规避贸易风险。

　　坚持高起点、高标准，建设好对外开放先导区。开发区要按照建设“新型工业化项目的集聚区、港口城市经济的示范区、对外开放的先导区、和谐社会的先行区”的要求，充分依托港口优势，全面提升城市功能，进一步扩大外引内联。全年吸引外商直接投资确保达到１．２亿美元，出口创汇５．６６亿美元。高新区要利用土地储备丰富和邻近主城区的优势，以开发高科技项目和培育产业集群为重点，确保主要经济指标实现倍增。

　　（五）继续实施“以港兴市”战略，加速沿海经济带建设

　　进一步完善港口基础设施，全面提高集疏运能力。以打造港口经济平台和建设服务东北的物流基地为目标，加速推进鲅鱼圈港口四期、航道改造、仙人岛港区等重点工程建设，积极改造营口老港区，全年实现固定资产投资２３亿元。着手谋划港前物流保税功能区建设，进一步完善港口信息服务系统，实施好“大通关”工程，提高港口管理水平和服务质量。强化货源开发，加大航线密度。全年完成港口吞吐量９０００万吨，集装箱运量１００万标箱。

　　积极推进沿海经济带开发，打造营口经济重要增长极。以营口港为辐射点，以沈大高速、哈大公路和滨海公路为框架，科学制定沿海１６００平方公里“金三角”经济区宏观规划。６月底前完成１２０平方公里辽宁（营口）沿海产业基地发展战略规划和４０平方公里起步区详规，当年实现基本建设投资１０亿元，确保完成土地回填１０平方公里。坚持“边建设、边招商”的原则，包装、储备、实施一批重点项目。同时，加快兴建仙人岛能源化工、冶金装备制造、镁质材料等各类园区，进一步发挥集聚效应和辐射作用。

　　（六）加快发展现代服务业，增强城乡综合服务功能

　　改造提升传统第三产业，大力发展新兴服务业。以大型专业市场为依托，优化城市商业布局，着力构建多层级的商品流通网络。积极推进农村现代流通体系建设，深入开展“万村千乡”试点工程，全年发展农村便民连锁店３００家。完善社区连锁和配送便民服务网络，努力实现社区服务产业化。加强市场监管，保障食品安全，维护正常的经济秩序。搞好企业信息化试点和社会公共领域信息化建设，加快发展法律、会计、保险、审计、公证、咨询等中介组织，努力为企业生产和群众生活服务。

　　积极培育现代物流业，强化物流服务功能。制定和完善现代物流业发展规划，研究出台优惠政策，建立促进发展的协调机制。加快营口港综合物流区、大石桥果品蔬菜批发市场等物流基地建设，积极发展原材料和商品批发、配送、运输和仓储服务物流企业。

　　以争创中国优秀旅游城市为契机，全面提高旅游业整体水平。进一步完善旅游业发展总体规划，整合旅游资源，积极开发海上旅游、温泉旅游和生态旅游，重点抓好西炮台遗址公园、鲅鱼圈月亮湖公园等旅游景点基础设施建设，办好第十二届望儿山母亲节。全年实现旅游业总收入１６．７亿元，增长１５％，力争年内进入中国优秀旅游城市行列。

　　（七）切实加强城乡规划、建设和管理，改善城市功能和人居环境

　　加强环境建设，提高城市品位。要按照争创国家卫生城和环保模范城的总体部署，完善“门前五包”责任制，深入开展爱国卫生运动，重点解决好小街小巷、非物业小区和城乡结合部环境脏乱差的问题，建立环卫保洁长效机制。严格实施环境评价和“三同时”制度，搞好城市烟尘、废气、噪声和固体废物的综合治理。以扩大城区绿地、提高绿化覆盖率为切入点，重点抓好辽河两岸、新建道路和住宅小区绿化，加强永远角区域生态保护，全年完成市区植树２０万株，新增绿地５０公顷。

　　提升城市整体功能，集中力量为群众办好实事。今年要以解决关系群众切身利益问题和促进社会事业发展为重点，努力办好１５件实事：（１）改造棚户区３０万平方米；（２）建设市区南部排涝工程；（３）改造市区供水管网１５０公里；（４）改扩建市区小街小巷道路９万平方米；（５）改造市区供热管网１００公里；（６）新建和改造环卫公厕８５座；（７）改造住宅小区７个；（８）改造河北街城区和渡口设施；（９）实施农村中小学现代远程教育工程；（１０）建设农村有线电视网；（１１）新建职业教育中心和实训基地；（１２）新建残疾人综合服务中心；（１３）新建食品检验中心；（１４）构建动物疫病防控体系；（１５）建设社会治安防控体系。同时，要切实抓好污水处理厂、垃圾处理厂、滨海热电厂二期、营柳路、庄林路等一批重点基础设施建设，力求早竣工、早受益。

　　提高城市规划管理水平，促进城市资产经营。认真落实城市总体规划，编制好分区规划、控制性规划，高起点、高标准做好开发区规划修编、小城镇规划完善和农民新村规划引导工作。强化规划管理，坚决查处违章乱建，维护规划的权威性和严肃性。加强建筑市场、城市出入口、城乡结合部等重点部位和重点地段的经常性管理，保持顺畅的交通秩序。加强社区建设与管理，充分发挥社区的作用。规范房地产开发和交易秩序，促进房地产市场健康发展。进一步完善土地收购储备和出让制度，提高国土资产收益水平。深化环卫、绿化、公交、水务等市政公用行业改革，努力实现投资多元化、运营市场化、发展产业化。

　　（八）切实做好财税金融工作，增强对国民经济和社会事业发展的支撑能力

　　进一步完善财政管理体制，确保财政收入稳定增长。规范财政收入级次，建立健全奖惩约束机制，促进增收节支，壮大县乡财源。加强税收和非税收入管理，完善税源动态监控体系，抓好重点财源，努力挖掘小税种的增收潜力。理顺财政体制，整合财力资源，壮大政府可控财力。转变财政对企业的支持方式，全面推行增值税转型改革。

　　坚持科学依法理财，充分发挥资金使用效益。深化部门预算、国库集中支付、政府采购、收支两条线等项改革，建立收入征收权、财力分配权、资金使用权、财政管理权相互制约机制。调整财政资金投向，重点向“三农”、社会保障和公益事业倾斜。加强市以下转移支付制度建设，制止税收返还等不规范的补助形式。大力压缩消费性支出，节约公用经费等项开支。加强政府债务管理，规范举债、担保行为，建立债务风险预警和偿债机制，防范和化解财政风险。

　　进一步优化金融环境，加大对经济发展的支持力度。积极推进城市商业银行增资扩股，支持各类商业银行处置不良资产和实施股份制改革。做好全省金融生态环境示范区的建设工作，改善企业融资环境。支持金融机构扩大信贷投放，创造条件吸引外埠银行进入我市。加强与国家开发行、农发行及其他股份制银行的合作，充分发挥中小企业（融资）服务中心、企业信用担保投资有限公司、中小企业信用建设促进会的作用，进一步完善中小企业融资服务体系。帮助企业做好股份制改造和上市辅导工作，完成港务股份股权分置改革。

　　（九）加强就业和社会保障工作，积极构建和谐社会

　　进一步拓展就业空间，努力扩大就业和再就业。继续把增加就业放在经济社会发展的突出位置，全面落实就业政策，重点帮扶“零就业家庭”、大龄就业困难群体和下岗失业人员，确保为每个有就业需求的“零就业家庭”至少提供一个就业岗位。全年新增实名制就业６．５万人。全面开展普惠制就业培训，提高就业人员职业技能和素质。进一步实施好农村劳动力转移工程，严厉查处拖欠农民工工资问题，改善农民进城务工环境。

　　积极规范社会保险制度，健全社会保障体系。以提率和扩面为重点，做好社会养老保险工作，确保基本养老保险个人帐户记清率和上解率达到１００％。实行养老保险市级统筹，加快企业退休人员社会化管理步伐。研究制订失地农民养老保险方案，维护农民合法权益。进一步健全农村医疗保险政策，完善城镇职工基本医疗保险制度，减轻参保人员负担。巩固城市低保成果，提高对重点地区、重点对象的救助水平，规范农村低保工作。

　　更加关心群众生活，解决好困难群体的实际问题。要不断探索有效的救助方式，加快建立对城乡特殊困难群众的社会救助体系。今年围绕解决特困群体生活困难，市政府将从以下三个方面出台帮扶政策。（１）建立城乡医疗救助体系，对城乡低保户、农村五保户、在乡老复员军人和７－１０级伤残军人等特殊困难群众实行应急医疗救助。（２）建立贫困家庭学生就学资助体系，由市、市（县）区政府投入和社会捐助筹集资金，对贫困家庭学生就学实行资助。（３）对棚户区改造安置中的特困户给予适当补贴，对特困家庭给予供暖补助。建立扶贫帮困长效机制，突出教育扶贫、科技扶贫、生产扶贫，着重增强其“造血”能力。

　　（十）积极发展科技、教育等项事业，促进社会全面进步

　　加快科技进步与创新，促进科技成果转化。完善支持企业自主创新的投入机制，尽快建立以企业为主体、市场为导向、生产与科研相结合的技术创新体系，加速科技成果转化和应用。加快科技兴企、科技兴农进程，逐步完善区域科技服务网络，全年引进农业新品种４０个，推广实用技术１５项。大力发展新材料和先进制造技术等领域的项目，加强知识产权保护，申报认定省级高新技术企业２０家，高新技术产业增加值确保增长３０％。

　　优先发展教育事业，促进人才培养和智力开发。坚持以满足群众教育需求为目标，认真落实“以县为主”的农村义务教育管理体制，逐步加大对农村教育的投入，促进城乡教育均衡发展。积极推进“双高普九”工作，提高普通高中的教育教学质量。继续实施素质教育，切实减轻中小学生课业负担，促进学生全面发展。按照区域化、特色化的原则，大力发展职业教育，加强省级标准化职教中心建设，积极开展订单教育和校企合作，调整和优化职业教育专业设置，提高办学质量和效益。重视发展高等教育，鼓励支持民办教育，形成各类教育共同发展的格局。加强师德师风建设，提高教师整体素质。努力构建现代国民教育体系，增强全民就业、创业和创新能力，建设学习型社会。进一步完善吸纳人才政策，建立和完善以教育培训为主导的人才培养机制、以市场为主导的人才配置机制和以利益分配为主导的激励机制，加速人才的引进、开发和培养，积极营造尊重劳动、尊重知识、尊重人才、尊重创造的社会环境，为人才搭建良好的创业平台。

　　重视文化、卫生、体育等项事业，促进经济社会协调发展。大力发展文化事业和文化产业，加强文物保护工作，繁荣和净化文化市场，活跃城乡文化生活。进一步完善公共卫生、疾病预防控制和医疗服务体系，提高重点公共卫生机构的技术装备水平、医疗服务水平和对突发公共卫生事件的应急处理能力。全面推进新型农村合作医疗工作，改善农村医疗卫生条件。坚持依法治育方针，实现由管理型向服务型的根本转变，新生婴儿性别比控制在正常值以内，人口自然增长率控制在１．８３‰。大力推进广电事业发展，积极拓展广播电视业务，不断提高节目编播质量。广泛开展全民健身运动，努力提高竞技体育水平。

　　加强思想道德建设，积极开展文明创建活动。强化思想政治工作，弘扬以爱国主义为核心的民族精神和以改革创新为核心的时代精神，激发全市人民热爱家乡、建设营口的热情。加强诚信教育，提高社会公信度。以未成年人为重点，加强公民道德教育，拓展精神文明创建载体，丰富创建内涵。以创建文明城市为龙头，推动文明村镇、文明社区、文明单位等群众创建活动再上新水平。

　　（十一）加强民主法制建设，全力维护社会稳定

　　发展社会主义民主，保障人民依法行使民主权利。自觉接受人大依法监督和政协民主监督，坚持向人大及其常委会报告制度，严格执行人大及其常委会的决议、决定，支持人民政协履行政治协商、民主监督、参政议政职能，认真办理人大代表建议和政协委员提案。不断完善民主决策机制，健全重大事项社会公示制度、听证制度和专家咨询制度，建立多种形式的决策咨询机制和信息支持体系。广泛听取民主党派、工商联、无党派人士的意见和建议，充分发挥工会、共青团、妇联等群团组织的桥梁和纽带作用。贯彻落实党的民族、宗教和侨务政策，增进社会和谐。

　　大力推进依法治市，维护社会稳定。全面开展“五五”普法教育和“四五”依法治市活动，促进各项工作依法管理。以打造“平安营口”为目标，严厉打击社会黑恶势力和各种刑事犯罪，加强社会治安综合治理，创造良好治安秩序，增强城乡居民的安全感。搞好国防教育、国防动员，加强民兵应急分队建设，做好社会拥军、科技拥军工作，争取再次进入全国双拥模范城行列。

　　强化安全生产监督管理，保障人民生命和财产安全。认真落实安全生产责任制，以矿山、石化、易燃易爆品等生产企业、重点建设项目、交通运输和人员密集场所为重点，搞好消防和安全生产专项整治，排查整改事故隐患，坚决遏制重特大事故发生。建立预警快速反应机制，增强应对自然灾害等重大突发事件能力。

　　切实做好信访工作，有效化解社会矛盾。认真落实《信访条例》，畅通信访渠道，不断完善各级领导信访接待日制度。坚持“属地管理，分级负责”的原则，落实信访工作责任制。综合运用政策、法律、经济、行政手段和教育、协商、调节等办法，依法及时合理地处理群众反映的问题，把矛盾化解在萌芽状态。

　　（十二）加强政府自身建设，努力创造良好的政务环境

　　强化民本意识，坚持执政为民。进一步增强群众观念，从群众最关心、最现实的问题入手，努力实现好、维护好、发展好人民群众的根本利益。树立正确的政绩观、权力观，敢于触及矛盾，善于破解难题，认真做好打基础、管长远的工作。认真贯彻《公务员法》，加强公务人员队伍建设，引导干部以超常规的思路谋划工作，用发展的办法解决问题。

　　转变政府职能，树立服务型政府形象。按照政企分开、政事分开的原则，进一步明确和规范政府行为，把政府工作的重点转到提供良好的公共服务上来，把政府经济管理的职能转到为市场主体服务和创造良好发展环境上来。全面落实国务院《依法行政实施纲要》，深入开展政务公开，建设公共行政服务中心，实施联合审批。落实行政执法责任制、过错追究制、绩效评估制，促进严格执法、文明执法、公正执法。积极推进事业单位机构和人事制度改革，力求做到创新体制，增强活力。

　　改进工作作风，大兴求真务实之风。坚持讲实话、办实事，克服形式主义、官僚主义作风。继续大力精简会议、文件，提高行政效能。大兴调查研究之风，倡导脚踏实地干事业的求真务实风气。着眼于解决具体问题，做艰苦细致的工作，切实把经济社会发展的各项工作落到实处。

　　坚持从严治政，进一步加强廉政建设。全面落实廉洁自律的各项规定，加强惩防体系建设，提高拒腐防变能力。坚决纠正损害群众利益的不正之风，充分发挥监察、审计、行政效能投诉中心等监督机构的作用，加强对行政机关运用权力、履行职责等情况的监督检查，以卓有成效的反腐倡廉成果取信于民。

　　各位代表，营口振兴，任务艰巨，责任重大，使命光荣。让我们在市委的正确领导下，在人大和政协的监督支持下，紧紧依靠全市人民的力量，坚定信心，抢抓机遇，埋头苦干，奋力开拓，确保圆满完成今年的各项任务，努力开创“十一五”营口经济社会快速发展的崭新局面！
